# The role of anticipation and neuroticism in developmental stuttering

**DOI:** 10.3389/fpsyg.2025.1576681

**Published:** 2025-05-21

**Authors:** Francesco Palombo, Francesca Del Gado, Francesca Rugolo, Stefano Lasaponara, Pierpaolo Busan, Donatella Tomaiuoli, David Conversi

**Affiliations:** ^1^Department of Psychology, La Sapienza University of Rome, Rome, Italy; ^2^Centro Ricerca e Cura, Rome, Italy; ^3^Department of Psychology, University of Turin, Turin, Italy; ^4^Department of Medical, Surgical, and Health Sciences, University of Trieste, Trieste, Italy; ^5^Tor Vergata University of Rome, Rome, Italy

**Keywords:** stuttering, anticipatory anxiety, DMN hypothesis, speech fluency, neuroticism, NEO-PI-3 scale, ASI-3 scale

## Abstract

**Purpose:**

People Who Stutter (PWS) are often characterized by the presence of cognitive-emotional issues, resulting in conditions such as social phobia and avoidance behaviors. Emotions have been demonstrated to have a role in modulating speech-motor systems. Thus, in PWS, emotion and cognition (i.e., higher levels of trait-stable-neuroticism-and contextual-anticipation-anxiety) could negatively influence speech-motor networks, resulting in an increased number of dysfluencies.

**Methods:**

To test this hypothesis, we recruited 13 PWS who were matched to 13 Fluent Speakers (FS). Participants were all Italian speakers and completed the NEO-PI-3 scale to assess neuroticism, and the ASI-3 scale for anxiety sensitivity. Successively, participants considered 55 words (repeated two times) and 55 sentences, and completed a task in which they had to evaluate their anticipation of stuttering before reading them aloud. Anticipation scores, reading times, and frequency of stuttering were evaluated and used for analyses.

**Results:**

Findings suggest that PWS mainly had higher social concern than the fluent speakers. Moreover, a tendency toward higher levels of neuroticism is evident. Linear regressions suggest that reading times in PWS (positively related to frequency of stuttering) may be mainly explained by stuttering anticipation scores and, secondarily, by neuroticism levels. Stuttering anticipation was also positively related to the recorded frequencies of dysfluencies.

**Conclusion:**

Stuttering anticipation and neuroticism may be useful indexes for predicting dysfluencies and speech behavior, in PWS. Surely, this may be related to long-life stuttering and adaptive/maladaptive compensation attempts. In every case, in a clinical context, this also suggests the importance of fully evaluating behavioral/emotional aspects of stuttering, to obtain a more complete picture of patients’ needs and “tailored”/multidisciplinary interventions.

## 1 Introduction

Developmental Stuttering (DS) is defined by the Diagnostic and Statistical Manual of Mental Disorders (Fifth Edition, DSM-5) of the American Psychiatric Association (APA), as a disturbance in the normal fluency and patterns of speech (American Psychiatric Association, 2013). DS is characterized by repetitions, prolongations, broken-up words, blocks, circumlocutions, and excessive physical tension. In addition, associated motor symptoms (i.e., blinking, tics, tremors, head shaking, breathing movements) may accompany dysfluencies. Stuttering usually appears in early childhood (i.e., 2–4 years), with a lifetime incidence of 5–8% (Yairi and Ambrose, 2013). However, only 1% will continue to persist in DS during adulthood (i.e., recovery rate of about 80%; Yairi and Ambrose, 2013). Importantly, etiologic and maintenance factors related to stuttering are still not completely understood.

DS has a likely multifactorial origin, often including genetic factors (e.g., Barnes et al., 2016; Frigerio-Domingues and Drayna, 2017; Frigerio-Domingues et al., 2019; Kraft and Yairi, 2012) that may modulate the appearance of a series of dysfunctions at a neural level. In fact, People Who Stutter (PWS) are usually characterized by impairments in sensorimotor networks useful for speech-motor planning and execution, involving brain regions such as the basal ganglia, the supplementary motor area, and the inferior frontal cortex (e.g., Alm, 2004; Busan, 2020; Chang and Guenther, 2020). Left hemispheric circuits are usually more affected than the right ones; in this context, networks of the right hemisphere could have a role in adaptive/maladaptive compensation of dysfluencies (e.g., Busan et al., 2019; Chang and Guenther, 2020; Etchell et al., 2018; Neef et al., 2018).

Importantly, DS has a series of consequences on behavior, emotions, and cognition (Alm, 2014). Compatibly, PWS may have moderately higher trait/state anxiety than fluent speakers (FS), likely driven by higher social anxiety (e.g., Alm, 2014; Craig and Tran, 2014). Negative correlations between state/trait anxiety and speaking rate may be also evident (Yang et al., 2017). In this context, emotions and cognition seem to be closely related: emotions influence thoughts, evaluations, and decision-making (Robinson et al., 2013). There is evidence that emotions affect several domains such as attention, motor skills, and language (Braine and Georges, 2023; Cunningham and Brosch, 2012; Gerritsen et al., 2008; Hinojosa et al., 2010, 2017; Rohr and Abdel Rahman, 2018). For example, stimuli with high emotional value may have a “priority” over neutral stimuli, capturing more attentional resources (Anderson and Phelps, 2001; De Martino et al., 2009; Peelen et al., 2009; Schwabe et al., 2011). This can result in cognitive biases: emotional stimuli that are irrelevant to the task can interfere with goal-directed behaviors, slowing reaction times and reducing response accuracies (Armony and Vuilleumier, 2013; Robinson et al., 2013). This is also true for speech and language: in healthy participants, Rohr and Abdel Rahman (2018) investigated the effect of “emotionally” charged words on different stages of language production, using electroencephalography (EEG). More specifically, cues with “negative” content were associated to an increase in “late positive potentials” in centro-parietal regions (associated with self-monitoring mechanisms). In addition, performance with “negative” stimuli was more error-rich than control tasks. Authors suggested that, for “negative” stimuli, heightened arousal was present (in comparison to “neutral” and/or “positive” cues). On turn, heightened arousal may interact with early stages of speech/language production, also due to an unbalanced focus on these cues.

Importantly, it has been suggested that internally-(vs. externally-)directed focus may contribute to disrupt simple/automatic movements (Kal et al., 2013; McNevin et al., 2003; Wulf et al., 2001), and this could be evident also in DS (e.g., Eichorn et al., 2016, 2019; Jackson et al., 2016).

Compatibly, one of the most interesting and controversial phenomena related to DS is stuttering anticipation (i.e., the ability to correctly predict and/or anticipate moments of dysfluencies). Anticipation is a “covert” phenomenon of stuttering (Jackson et al., 2018), and awareness of being about to stutter is often related to the activity of the autonomic nervous system and/or to feelings of anxiety (Alm, 2014; Bowers et al., 2012; Drabant et al., 2011; Rodgers and Jackson, 2021). In fact, anticipation could be like an “alarm” bell that the brain would learn and exploit, trying to avoid dysfluencies (see Garcia-Barrera and Davidow, 2015). Different studies already tried to investigate the possible relationships between anticipation and stuttering. For example, Brocklehurst et al. (2012) showed that participants were able to anticipate when they would stutter, but this evidence was influenced by feedbacks on speech fluency, even when not consistent with the real performance. This may suggest that communication failures may exacerbate stuttering symptoms. On this line, Bowers et al. (2012) tested the possibility that stuttering anticipation may correlate with autonomic arousal and frequency of dysfluencies. Findings showed that a “solo *vs.* chorus” condition induces an increase in arousal parameters and a greater amount of dysfluencies. Compatibly, Arenas and Zebrowski (2017) showed that anticipation is a common characteristic of PWS and that it correlates with stuttering severity, with the evidence of consistency among anticipation scores reported in different evaluation settings. All this considered, we can hypothesize that PWS’ characteristics related to anticipation, autonomic arousal, and anxiety may be “grouped” by particular personality factors, such as neuroticism (e.g., Bleek et al., 2012; Iverach et al., 2010; Montag et al., 2012). Neuroticism is a personality trait characterized by elements such as anxiety, anger, depression, self-awareness, impulsivity, withdrawal, volatility, and/or vulnerability. This trait may predict emotional reactivity, i.e., the degree and manner in which a person reacts to specific stimuli, especially to the “negative” ones (Robinson et al., 2013). Accordingly, individuals with high neuroticism levels are more likely to change their attitude following errors and are more sensitive to “negative” feedbacks (Robinson et al., 2013).

All this considered, we hypothesize that factors such as neuroticism and anxiety sensitivity may be in relation with speech performances in PWS. More specifically, higher levels of neuroticism and anxiety should result in higher reading times and dysfluencies (as measured in the present work). Consequently, this study aims to investigate whether (i) neuroticism, together with (ii) anxiety sensitivity (specifically in the “social concern” subscale), and (iii) stuttering anticipation levels may predict speech reading times (and, also, frequency of dysfluencies) in PWS. For this reason, we also investigated if some differences exist in values of neuroticism and anxiety sensitivity between PWS and normative samples, and if some differences could be evident when considering successive readings by PWS (as well as when comparing PWS and the control group). Finally, we tested the possible existing correlations between ratings of anticipation in PWS, and correlations between anticipation, reading times, and frequency of dysfluencies in the same group.

## 2 Materials and methods

### 2.1 Participants

Thirteen PWS (6 females; age range 24–50 years; mean = 31.5 years-SD ± 7.41-; continuous years in education: mean = 16.69 years -SD ± 2.18-) were recruited and compared to 13 matched Fluent Speakers (FS; 6 females; age range 20–62 years; mean = 36.85 years -SD ± 15.74-; continuous years in education: mean = 15.54 years, -SD ± 2.5-). Participants of the PWS group were affected by DS from childhood, with no reported past or present comorbidities. FS were matched to PWS for age, education, and sex, and they reported no past or current stuttering. Two-sample *t*-tests indicated that age and education were comparable between PWS and FS (age, *p* = 0.28; education, *p* = 0.22). None of the participants reported having previous or current neurological or neuropsychiatric disorders (besides stuttering in the PWS group), also related to other speech, language, and/or learning disorders. All participants were native Italian speakers.

PWS were recruited by convenience, thanks to the help of “CRC Balbuzie” (Rome, Italy). Also FS were recruited by convenience, mainly exploiting word of mouth procedures starting from “La Sapienza” University (Rome, Italy). All PWS reported previous participation in stuttering courses/therapy/rehabilitation at least once during their lifetime. This study was approved by the Ethics Committee of the Faculty of Psychology, “La Sapienza” University, Rome, Italy (authorization number: 0001141) and was in accordance with principles described in the “Declaration of Helsinki.” Every participant signed an informed consent, and were allowed to leave the study at any moment. No compensation was provided for participation in the study.

### 2.2 NEO-PI-3

The NEO-PI-3 scale (McCrae et al., 2005) is a 240-item questionnaire that assesses 30 specific facets, 6 for each of five basic personality dimensions (*Big Five* model): Neuroticism (N), Extraversion (E), Openness to Experience (O), Agreeableness (A), and Conscientiousness (C). Items are answered on a 5-point Likert scale, ranging from “strongly disagree” to “strongly agree.” In about 35–45 min, the NEO-PI-3 provides a systematic assessment of emotional, interpersonal, experiential, attitudinal, and motivational styles.

### 2.3 ASI-3

The Anxiety Sensitivity Index (ASI-3; Reiss et al., 1986) is a 16-item measure on which respondents indicate the degree to which they fear the potential “negative” consequences of anxiety-related symptoms. The ASI-3 includes a “physical concerns” factor (PC; 8 items), a “social concerns” factor (SC; 4 items) and a “cognitive concerns” factor (CC; 4 items). Participants are asked to indicate the extent to which they agree or disagree with each item on a 5-point Likert scale (0 = “very little” to 4 = “very much”).

### 2.4 Experimental task

First, participants were administered the above described standardized questionnaires (with availability of Italian norms; see Fossati and Ciancaleoni, 2014; Costa et al., 2006; Petrocchi et al., 2014) to assess: (i) participants’ personality scores (NEO-PI-3), and (ii) anxiety sensitivity (ASI-3). Besides that, they were presented with a list of words (50 items) that were all taken from “The New Basic Vocabulary of the Italian Language” (De Mauro, 2016; see Supplementary Figure S1). Words were selected for frequency of use (the chosen vocabulary is composed by the most frequently used words in Italian), representativity (i.e., all the alphabet letters were used in the initial phoneme, except x/y/k), complexity, and length (i.e., 3–7 letters, corresponding to 2–4 syllables). Difficulty of reading was also considered (i.e., participants with stuttering were requested to write down several words that were difficult for them to read; successively, in line with the selection criteria, 5 of them were added to the sample -i.e. a total of 55 words were available-). PWS had to evaluate the likelihood of stuttering for each word by using a VAS scale (range 0–5 points; higher likelihood = higher score), answering the question “How much do you feel/think you would stutter reading this word aloud?” (“pre-task” anticipation).

**Figure 1:**
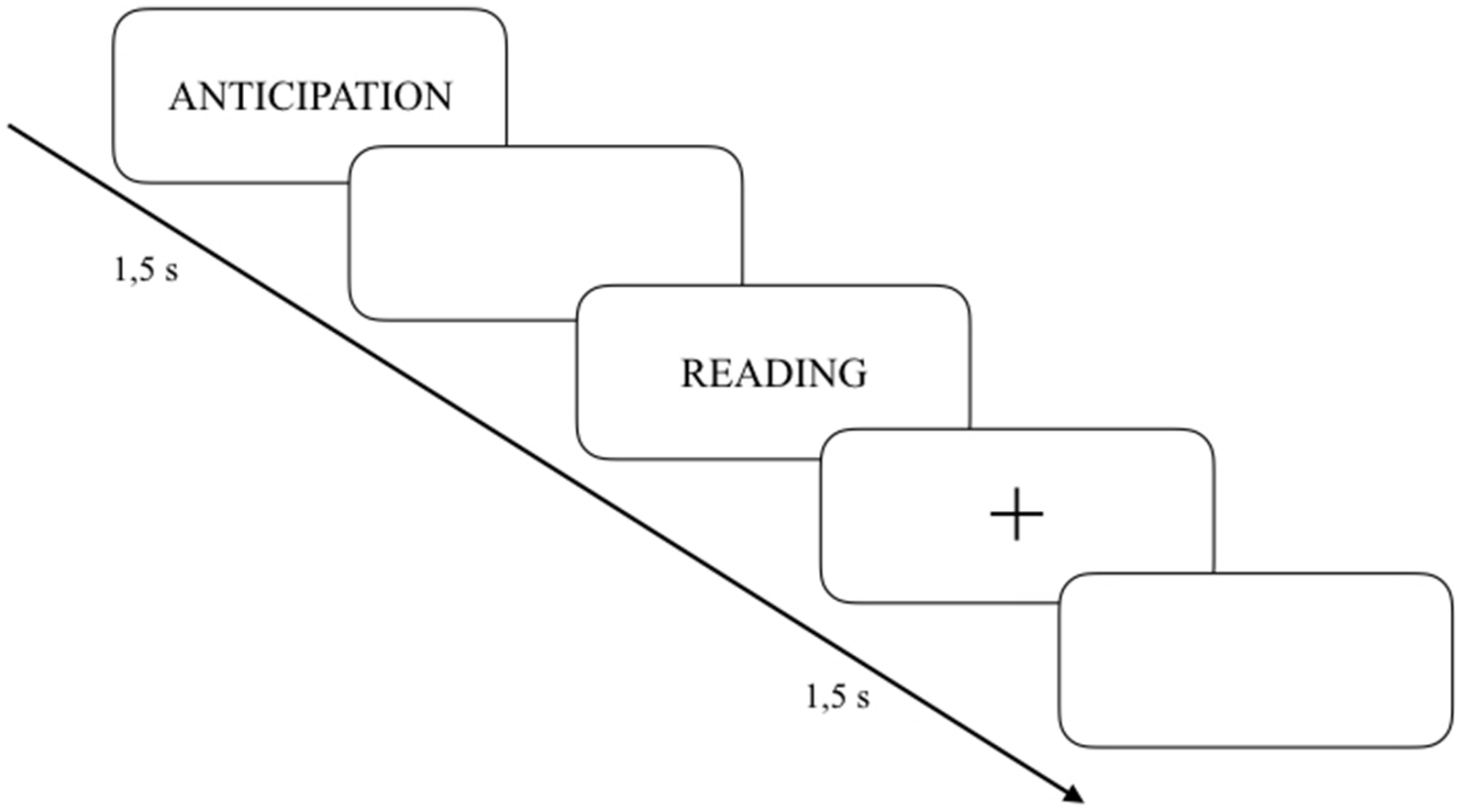
Schematic representation of the experimental task proposed to participants.

Successively, in a second session (i.e., during the experimental task), PWS were again requested to anticipate the likelihood of stuttering, this time just before reading aloud the same words (VAS scale, range 0–5 points; higher likelihood = higher score). In this last case, once answered (and after a delay period of 1,500 ms), they were allowed to read them aloud. After that, using a fixation point (and a further 1,500 ms delay), the same words were randomly repeated (with the same procedures) singularly and in sentences including them (see Supplementary Figure S2). In conclusion, a total of 55 (50 + 5 chosen by participants) items were repeated 3 times to form 110 stimuli (i.e., 55 words, repeated 2 times) and 55 sentences to be read. Sentences were selected to reflect common and everyday language use. For example, many phrases related to daily activities such as asking for water, going to the bank, getting a haircut, or having breakfast. Colloquial and idiomatic Italian language was also considered. Finally, in the context of lexical and syntactic accessibility, sentence structures were always comprehensible and aligned with conversational Italian language, especially in their length (mean = 16.07 syllables -SD ± 2.8-). All procedures were computerized by using the software Testable (Rezlescu et al., 2020), and a trial session (7 words and 7 phrases containing those words) was allowed for familiarization with the task. The total duration of the experiment was about 35 min (see Figure 1). The following indexes were calculated: stuttering anticipation scores (recorded in a “pre-task” mode -i.e. before the task-and during the task -i.e. “word 1” anticipation scores and “word 2” anticipation scores-), reading times, and frequency of dysfluencies (obtained from audio-visual recordings during tasks).

Please note that this experimental task was inspired by Arenas and Zebrowski (2017), in which PWS had to evaluate twice (i.e., during two visits) the stuttering anticipation level of 50 words, complete an anticipation questionnaire, and read the 50 words in a loud and “fast” way. In addition, in the present work we included a rating of the anticipation scores obtained during the experimental task (i.e., just before reading), sentence reading, and the use of NEO-PI-3 and ASI-3 questionnaires.

### 2.5 Data acquisition

Reading times were considered starting from stimulus presentation until the complete word has been read (ms). Reading times were calculated (i) at the first appearance of the word; (ii) at the second appearance of the word, and (iii) at the appearance of the sentence. Data analysis was realized by using Audacity® (an open-source audio editing software, version 3.4.2; Audacity Team, 2014), in conjunction with video recordings to facilitate calculations. Similar data were also obtained in fluent participants, who read all the words and sentences used for PWS (and using similar procedures) to compare reading times between groups.

Stuttering episodes (frequency of dysfluencies, calculated as the percentage of stuttered syllables) were evaluated considering interruptions of the normal/rhythmic flow of speech, also considering motor/gestural and/or linguistic expression (e.g., blocks, repetitions, prolongations, motor gestures of rigidity, assessed on each syllable; the maximum score on each syllable is 1). Stuttering episodes were considered (i) at the first appearance of the words, (ii) at the second appearance of the words, and (iii) during the reading of the sentences.

### 2.6 Statistical analysis

Statistics were performed using Jamovi (Navarro and Foxcroft, 2019). More specifically, when considering differences in the NEO-PI-3 scales, unpaired t-tests were estimated between the 13 PWS participants and a normative sample, constituted of 569 Italian subjects (Costa et al., 2006). When considering the ASI-3 questionnaire, the three subscales (SC, PC and CC) were analyzed in a similar manner using a unpaired t-test and comparing the 13 PWS participants to a normative sample (constituted of 629 Italian subjects; Petrocchi et al., 2014) and a clinical sample (constituted of 129 Italian subjects; Petrocchi et al., 2014).

Successively, based on the results, we evaluated the influence of neuroticism (as well as the influence of factors composing this NEO-PI-3 score such as subscales of self-consciousness and depression), social and cognitive concern (ASI-3; SC and CC), and stuttering anticipation on reading times through three different multiple linear regression models that were performed on data obtained in experimental participants (PWS). In this case, reading times were considered and separately averaged (for each participant) in the three different conditions (i.e., “word 1” presentation, “word 2” presentation, and “sentence” presentation). In addition, stuttering anticipation data (considering values obtained during “pre-task” evaluation and during task execution) were separately averaged and calculated for every condition. Please note that, when considering sentence reading, anticipation data were not acquired: as a consequence, for regression analysis, we considered an “average” value obtained from measurements recorded at “word 1” and “word 2” presentation. Frequencies of dysfluencies were also computed for each participant and separately calculated in the three conditions. In conclusion, in the three multiple linear regressions, neuroticism data, social and cognitive concern subscales, and anticipation data related to the tasks were considered as independent variables, while reading times were considered as the dependent variable.

After that, the reading times of the three conditions (“word 1,” “word 2,” and sentences) were separately compared between PWS participants and the control group (two-sample t-test). A paired-sample t-test was also performed comparing reading times at “word 1” presentation and reading times at “word 2” presentation in PWS (and controls), as well as comparing frequencies of dysfluencies obtained at “word 1” and “word 2” presentation.

Before performing regression analysis and t-tests, statistical validity assumptions (such as autocorrelation, collinearity, normality, and residual plots) were verified. Effect sizes (Cohen’s *d*; see Lenhard and Lenhard, 2022) were also calculated and reported, when appropriate.

Finally, correlations (Pearson’s correlation) were performed considering stuttering anticipation data obtained in the “pre-task” (i.e., before the task) and anticipation data obtained during task execution (“word 1” anticipation and “word 2” anticipation, separately calculated), as well as considering stuttering anticipation data and frequencies of dysfluencies in the three different conditions. Frequencies of dysfluencies were also correlated with the correspondent reading times.

Considering the exploratory nature of this work, raw (i.e., not corrected) statistical values are reported. Similarly, significance was always stated at *p* < 0.05 (while *p* < 0.1 was considered as a statistical trend).

## 3 Results

### 3.1 NEO-PI-3

**Figure 2:**
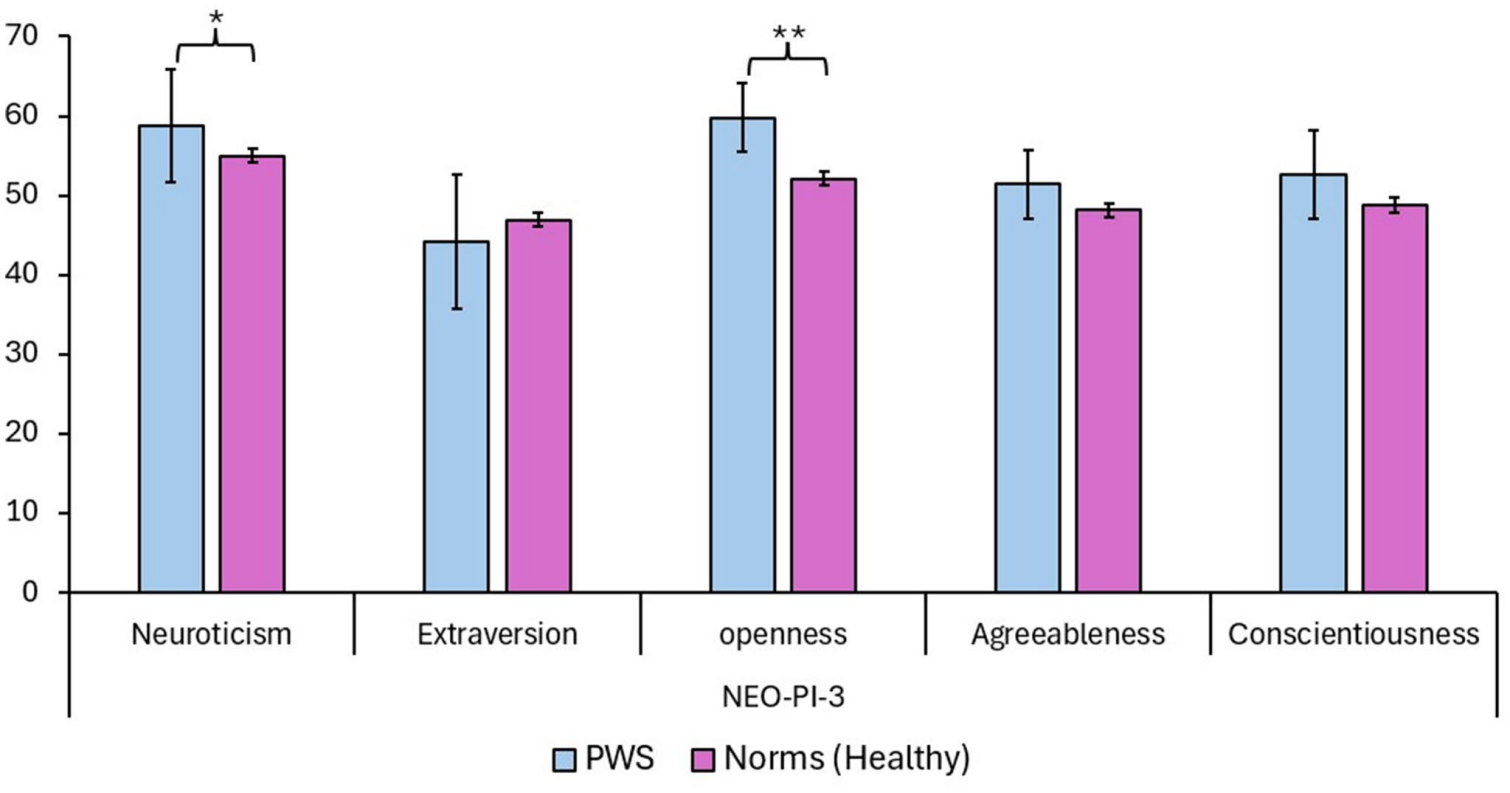
Standardized PWS scores of the NEO-PI-3 scales are shown in comparison to data obtained from NEO-PI-3 norms. Asterisks are used for reporting significant differences among comparisons (* = statistical trend, *p* < 0.1; ** = *p* < 0.05). Data are reported as means and with confidence intervals.

The averaged and standardized t-score of the 13 PWS participants who completed the NEO-PI-3 questionnaire resulted in a statistical trend (*p* = 0.09; small effect size, Cohen’s *d* = 0.32) for the Neuroticism scale (compared to values obtained from an Italian normative sample; Costa et al., 2006), suggesting higher levels of Neuroticism in PWS. Also, the Openness scale resulted in a significant difference in PWS (*p* = 0.0031, compared to the normative sample; large effect size, Cohen’s *d* = 0.85), indicating higher values in the group of PWS. However, considering the aim of the present work, we were more interested in findings related to possible “negative” and/or “detrimental” aspects that could have an effect on stuttering, and thus only Neuroticism was considered for further analysis. As a consequence, we also qualitatively evaluated the Neuroticism subscales that had at least 1 deviation standard difference when compared to the normative sample (see Fossati and Ciancaleoni, 2014): in this case, findings suggest the presence of higher scores in PWS for the depression (PWS, mean = 60.23, SD = ±14.04) and the self-consciousness (PWS, mean = 61.77, SD = ±12.83) subscales. Data are summarized in Table 1 and in Figure 2.

**Table 1:** PWS scores on the NEO PI-3 scales are shown in comparison to Italian norms (Costa et al., 2006).

Scale	Participants	Mean (±SD)	Confidence intervals (95%)	*p-*value
Neuroticism	People Who Stutter	58.77 (±13.23)	51.58/65.96	0.09*
	Norms	55 (±10.1)	54.17/55.83	
Extraversion	People Who Stutter	44.08 (±15.54)	35.63/52.52	N.S
	Norms	46.9 (±10.9)	46/47.8	
Openness	People Who Stutter	59.77 (± 8,02)	55.41/64.13	0.0031**
	Norms	52.1 (±10.0)	51.28/52.92	
Agreeableness	People Who Stutter	51.38 (±7.94)	47.06/55.7	N.S.
	Norms	48.1 (±11.0)	47.2/49	
Conscientiousness	People Who Stutter	52.62 (±10.42)	46.96/58.28	N.S.
	Norms	48.7 (±11.6)	47.75/49.65	

Only significant (**, in bold) or statistical trend (*, in italics) *p*-values are reported.

### 3.2 ASI-3

**Figure 3:**
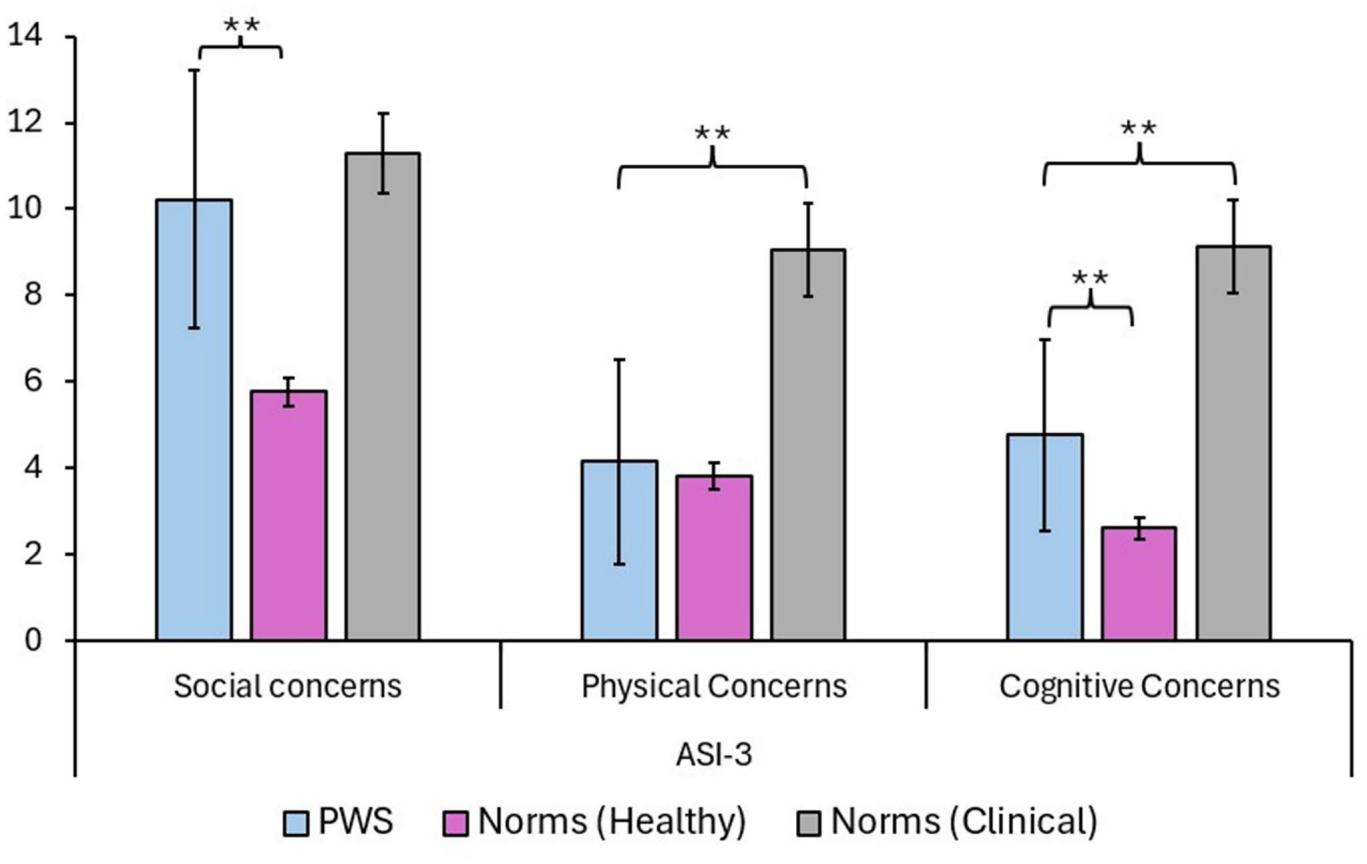
Findings of the PWS group at the ASI-3 questionnaire are divided into the three subscales Social Concerns (SC), Physical Concerns (PC), and Cognitive Concerns (CC). Data were compared to the normative results of healthy and clinical samples (Petrocchi et al., 2014). Findings mainly show that PWS reports higher scores in the SC subscale and the CC subscale with respect to the non-clinical sample. Importantly, SC scores are comparable to those of the clinical sample. Asterisks are used for reporting significant differences (*p* < 0.05) among comparisons. Data are reported as means and with confidence intervals.

The ASI-3 questionnaire resulted in significant differences in PWS (compared to the normative sample; see Petrocchi et al., 2014) when considering the SC subscale (*p* < 0.001; large effect size, Cohen’s *d* = 0.93) and the CC subscale (*p* = 0.007; medium effect size, Cohen’s *d* = 0.6). More specifically, PWS resulted in higher levels of social and cognitive concerns compared to the non-clinical population. No differences were evident when considering the PC subscale. Similarly, when comparing PWS to normative data obtained from clinical samples (Petrocchi et al., 2014), PWS resulted in significantly lower levels of CC (*p* = 0.024; large effect size, Cohen’s d = 0.78) and PC (*p* = 0.012; large effect size, Cohen’s *d* = 0.85), but not SC (*p* = 0.53). In conclusion, PWS seemed to be strongly affected at the SC level. Data are summarized in Table 2 and Figure 3.

**Table 2:** PWS scores on the ASI-3 scales are shown in comparison to Italian norms (Petrocchi et al., 2014).

Scale	Participants	Mean (±SD)	Confidence intervals (95%)	*p-*value
Social concerns	People Who Stutter	10.23 (±5.46)	7.26/13.2	<0.001** *(vs. Healthy)*
	Norms (Healthy)	5.77 (±4.03)	5.46/6.09	
	Norms (Clinical)	11.3 (±5.85)	10.38/12.22	
Physical concerns	People Who Stutter	4.15 (±4.34)	1.79/6.51	0.012** (vs. Clinical)
	Norms (Healthy)	3.82 (± 4.02)	3.51/4.13	
	Norms (Clinical)	9.04 (±6.84)	7.96/10.12	
Cognitive concerns	People Who Stutter	4.77 (±4.07)	2.56/6.98	0.007** (vs. Healthy) 0.024** (vs. Clinical)
	Norms (Healthy)	2.60 (±3.15)	2.35/2.85	
	Norms (Clinical)	9.14 (±6.79)	8.07/10.21	

Only significant (**, in bold) or statistical trend (*, in italics) *p*-values are reported.

### 3.3 Experimental task

Reading times of PWS and the control group are reported in Table 3 (“word 1” presentation, “word” 2 presentation, and sentence reading). Stuttering anticipation scores and frequencies of dysfluencies in the three different conditions are also reported.

**Table 3:** Reading times are reported in seconds.

Indexes/Group	People who stutter	Control group	Confidence intervals (95%)
Reading times (word 1)	1.49 (±0.98)	0.64 (±0.09)	PWS: 0.96/2.02 CG: 0.59/0.69
Reading times (word 2)	1.43 (±0.95)	0.63 (±0.09)	PWS: 0.91/1.95 CG: 0.58/0.68
Reading times (sentences)	5.38 (±4.49)	2.52 (±0.28)	PWS: 2.94/7.82 CG: 2.37/2.67
Stuttering anticipation (word 1)	1.86 (±0.85)	NA	PWS: 1.4/2.32 CG: NA
Stuttering anticipation (word 2)	1.64 (±0.79)	NA	PWS: 1.21/2.07 CG: NA
Stuttering anticipation (sentences)	1.75 (±0.81)	NA	PWS: 1.31/2.19 CG: NA
Frequency of dysfluencies (word 1)	9.8% (±18%)	NA	PWS: 0.02/19.59% CG: NA
Frequency of dysfluencies (word 2)	9.3% (±18%)	NA	PWS: 0/19.09% CG: NA
Frequency of dysfluencies (sentences)	6.8% (±14%)	NA	PWS: 0/14.41% CG: NA

Stuttering anticipation scores are reported on a VAS scale, ranging 1–5. Frequencies of dysfluencies are calculated considering elements such as blocks, repetitions, and prolongations on each syllable and are reported in percentages. Data are reported as means (±SD) and with confidence intervals.

A T-test performed on reading times at “word 1” presentation revealed a significant difference between PWS and the control group (*p* = 0.002; large effect size, Cohen’s *d* = 1.21; PWS slower than controls). Similarly, we observed a significant difference in reading times at “word 2” presentation (*p* = 0.003; large effect size, Cohen’s *d* = 1.19; PWS slower than controls) and in reading times of sentences (*p* = 0.015; large effect size, Cohen’s *d* = 0.899; again, PWS slower than controls). Then, we investigated if reading times and dysfluencies were affected by the tasks (i.e., word presentation), in both groups. In PWS, findings suggest a significant difference when comparing reading times of “word 1” vs. reading times of “word 2” (*p* = 0.038; medium effect size, Cohen’s *d* = 0.65; PWS slower at “word 1” presentation), while no differences were evident in the control group (*p* = 0.78). Frequencies of dysfluencies (PWS; “word 1” presentation vs. “word 2” presentation) were not statistically different (*p* = 0.396).

Successively, we tried to relate neuroticism scale findings (NEO-PI-3 questionnaire; also considering depression and self-consciousness subscales), SC and CC subscales findings (assessed by means of the ASI-3 questionnaire), and stuttering anticipation values with reading times obtained in PWS (and separately assessed in the three different conditions: “word 1” presentation, “word 2” presentation, and sentence presentation).

More specifically, when considering the first multiple linear regression analysis (“word 1” presentation), the model is significant (*p* = 0.034) allowing to explain 83.6% of the variance (*R*^2^; see Table 4).

**Table 4:** *R*-value and *R*^2^ of the first multiple linear regression analysis (i.e. data obtained at the first presentation of the single word).

Model fit measures							
Model	*R*	*R* ^2^	Adjusted *R*^2^	Overall model test			
				*F*	df1	df2	*P*
1	0.914	0.836	0.673	5.11	6	6	0.034

Omnibus ANOVA test					
Variable	Sum of squares	df	Mean square	*F*	*P*
Self-consciousness scale	5.78e-4	1	5.78e-4	0.00183	0.967
Depression scale	0.971	1	0.971	3.06629	0.130
Cognitive concerns	0.792	1	0.792	2.50098	0.165
Social concerns	1.077	1	1.077	3.40067	0.115
Neuroticism	1.354	1	1.354	4.27792	0.084
First word anticipation	7.300	1	7.300	23.06008	0.003
Residuals	1.899	6	0.317		

Model coefficients are also reported. Type 3 sum of squares.

Model coefficients show that only the main factor related to the independent variable of stuttering anticipation is significant (*p* = 0.003). A statistical trend for the neuroticism factor (*p* = 0.084) is also evident. This means that, in PWS, reading times may be “impacted” by stuttering anticipation and, partially, by neuroticism levels.

Similarly, the second multiple regression analysis performed on “word 2” presentation shows that the model is significant (*p* = 0.008), managing to explain 90.3% of the variance (see Table 5). Again, findings suggest that stuttering anticipation can significantly modulate reading times of PWS (*p* < 0.001).

**Table 5:** Findings show the *R*-value and *R*^2^ of the second multiple linear regression analysis (i.e. data obtained at the second presentation of the single word).

Model fit measures							
Model	*R*	*R* ^2^	Adjusted R^2^	Overall model test			
				*F*	df1	df2	*P*
1	0.95	0.903	0.806	9.33	6	6	0.008

Omnibus ANOVA test					
Variable	Sum of Squares	df	Mean square	*F*	*P*
Self-consciousness scale	0.0288	1	0.0288	0.164	0.699
Depression scale	0.2939	1	0.2939	1.680	0.243
Cognitive concerns	0.0690	1	0.0690	0.395	0.553
Social concerns	0.4708	1	0.4708	2.691	0.152
Neuroticism	0.4671	1	0.4671	2.670	0.153
Second word anticipation	7.2101	1	7.2101	41.211	< 0.001
Residuals	1.0497	6	0.1750		

Model coefficients are also reported. Type 3 sum of squares.

Finally, the third multiple regression analysis performed on sentence presentation shows that the model is significant (*p* = 0.034) managing to explain 83.6% of reading times variance, in PWS (Table 6). In this case, not only stuttering anticipation is significant in modulating responses (*p* = 0.002), but also the depression subscale (*p* = 0.036) and the neuroticism scale (*p* = 0.029) resulted as possible modulators of reading times variability in PWS. A statistical trend (*p* = 0.098) was also evident for CC.

**Table 6:** Findings show the *R*-value and *R*^2^ of the third multiple linear regression analysis (i.e., evaluating reading times at “sentence” presentation).

Model fit measures							
Model	*R*	*R* ^2^	Adjusted *R*^2^	Overall model test			
				*F*	df1	df2	*P*
1	0.915	0.836	0.673	5.11	6	6	0.034

Omnibus ANOVA test					
Variable	Sum of squares	df	Mean Square	*F*	*P*
Self-consciousness scale	6.03	1	6.03	0.912	0.377
Depression scale	48.01	1	48.01	7.259	0.036
Cognitive concerns	25.26	1	25.26	3.820	0.098
Social concerns	10.17	1	10.17	1.537	0.261
Neuroticism	53.54	1	53.54	8.095	0.029
Sentences anticipation	179.30	1	179.30	27.111	0.002
Residuals	39.68	6	6.61		

Model coefficients are also reported. Type 3 sum of squares.

### 3.4 Correlations

Analyses showed the presence of a positive correlation between stuttering anticipation and frequencies of dysfluencies, in the three different tasks. More specifically, this was evident when considering anticipation values of “word 1” presentation and the correspondent frequencies of dysfluencies (*r* = 0.73, *p* = 0.005). Similarly, when considering “word 2” presentation, anticipation and dysfluencies resulted in a correlation value of *r* = 0.877 (*p* < 0.001). Finally, when considering anticipation values estimated for the sentence task and the corresponding amount of dysfluencies, correlation resulted in a value of *r* = 0.71 (*p* = 0.006).

When considering correlations between stuttering anticipation scores evaluated before tasks execution and the “effective” anticipation perceived during words presentation, data suggests the presence of a significant and positive correlation during the different phases of the experiment (i.e., “word 1” and “word 2”; *r* = 0.948, *p* < 0.001). On the other hand, non-significant correlations were highlighted with “pre-task” evaluations (“word 1”: *r* = 0.325, *p* = 0.278; “word 2”: *r* = 0.309, *p* = 0.304).

Finally, when considering correlations between reading times and frequencies of dysfluencies (evaluated in the three different tasks), data are highly and positively correlated (“word 1”: *r* = 0.943, *p* < 0.001; “word 2”: *r* = 0.949, *p* < 0.001; sentences: *r* = 0.983, *p* < 0.001).

## 4 Discussion

In the present work, PWS resulted in higher levels of neuroticism, SC and CC when compared to normative samples (please note that, in PWS, CC resulted less affected than “clinical” sample). However, only the former (especially during sentences reading) and stuttering anticipation scores (in all the conditions), were good predictors of reading times in PWS, suggesting that behavioral/emotional factors could be useful for predicting speech performances in stuttering. In addition, reading times of PWS were significantly slower than those of the control group (obviously, this effect could be due to the presence of dysfluencies in PWS, as suggested by positive significant correlations between reading times and frequencies of dysfluencies, in the three different conditions; compatibly, stuttering anticipation scores were positively related to frequencies of dysfluencies).

All this considered, and also on the basis of previous research (e.g., Bleek et al., 2012; Hedinger et al., 2021; Iverach et al., 2010; Montag et al., 2012), the present work suggests that, in PWS, higher-than-average neuroticism (thus, also taking in account scores obtained at the depression and self-consciousness subscales of the NEO-PI-3 questionnaire) could be considered, along with factors of stuttering anticipation, as good predictors and/or modulators of speech performance (i.e., increased reading times; anxiety sensitivity -i.e. subscales of the ASI-3 questionnaire-seems to have less effect in this context). More specifically, as the difficulty of the task increases, peculiarities arise in the data: whereas in the single-word reading tasks the only significant predicting variable is stuttering anticipation, in the sentence reading tasks the variables of neuroticism and depression subscale also influence PWS’ speech performance.

Various mechanisms could be hypothesized to modulate this process, which will be expanded in the next sections.

### 4.1 The role of anticipation in stuttering

Using regression analysis, it was shown that the greatest part of variance of speech performance in PWS may be in relation with evaluation of stuttering anticipation, confirming previous evidence highlighting the importance of this construct in stuttering (Arenas and Zebrowski, 2013; Brocklehurst et al., 2012; Garcia-Barrera and Davidow, 2015; Jackson et al., 2018).

Anticipation could be described as a precipitating factor for stuttering (see Arenas, 2017; Bloodstein, 1975; Brocklehurst et al., 2013; Garcia-Barrera and Davidow, 2015; Jackson et al., 2018, 2022). Compatibly, anticipation scores and frequencies of dysfluencies resulted here as strongly correlated, also proving that anticipation scores may predict the observed behavior on reading times. Stuttering anticipation may be described as an “alarm bell” that could help or disrupt speech fluency (Garcia-Barrera and Davidow, 2015) because it allows to use strategies to avoid blocks (i.e., trying to speak fluently) but it can also precipitate worry and fear of speech situations. On this line, Jackson et al. (2018) outlines that almost every PWS can use some strategies to change their speech production in response to anticipation (for example, circumlocutions and/or substitutions, changing speech rate, using pseudo-stuttering, etc.). On the other hand, Briley (2023) suggest that anticipation should be more properly described as a stuttering moment, i.e., a “covert” stuttering: in this case, it’s not important if the person stutters or not, because when the person experiences blocks and/or anticipation (consciously or not), the consequence of that perception will be an alteration of the normal act of speaking.

In this context, previous evidence in neuroscience could help in clarify stuttering anticipation mechanisms. Research has shown the involvement of (not only) sensory-motor areas, but also of brain regions related to monitoring, emotional and cognitive abilities. In this context, Jackson et al. (2022) observed that stuttering anticipation is related to the consistent over-activation of the right prefrontal cortex, thus possibly interacting with neural systems that are part of the Default Mode Network (DMN; Chang et al., 2018; Ghaderi et al., 2018; Gracco et al., 2022). The prefrontal cortex is part of the DMN, i.e., a group of mutually interconnected brain regions (comprising nodes such as the inferior parietal cortex and the posterior cingulate cortex; see Andrews-Hanna et al., 2014) characterized by high basal (i.e., at “rest”) metabolism and perfusion (Raichle, 2015). On the other hand, DMN tends to show lower levels of activity when the brain is actively involved in “goal-oriented” tasks (Andrews-Hanna et al., 2014; Raichle, 2015). More specifically, the DMN shows anti-correlations with task-positive networks like those supporting attention, executive control, and somato-motor functions (compare with Garnett et al., 2022; Menon, 2023). Compatibly, it has been suggested that performing “fluid” and/or automatic motor tasks can easily “break down” when attention is focused inwardly on oneself (thus, more linked to DMN) versus outwardly or toward a movement target (compare with Garnett et al., 2022). This was also shown in dual-task conditions (see Eichorn et al., 2016, 2019), in which attention was manipulated and allocated away from speaking during a conversation. In conclusion, internally-(vs. externally-) directed focus may contribute to disrupt simple/automatic movements (see for example Kal et al., 2013; McNevin et al., 2003; Sonuga-Barke and Castellanos, 2007; Wulf et al., 2001), also in stuttering (compare with Eichorn et al., 2016, 2019; Jackson et al., 2016).

Interestingly, DMN has been suggested to have a role in negative reactions to stuttering, thus likely contributing to the development of social anxiety (Alm, 2014; see also Pasculli et al., 2024). As in a vicious circle, this anxiety is reinforced by the anticipation of stuttering (Jackson et al., 2015, 2018, 2019; Rodgers and Jackson, 2021), thus possibly resulting in repetitive/negative thinking or rumination (Tichenor and Yaruss, 2020) and, therefore, in higher neuroticism. Interestingly, Orpella et al. (2024) suggest that this may result in a further abnormal involvement of neural control on speech production processes, especially before an anticipated and/or stuttered (vs. fluent) speech (Jackson et al., 2022). More specifically, a hyperactive control mechanism (and/or the excessive motor inhibition than can result from it) seems to negatively modulate neural activity of the right pre-supplementary motor area, especially before speech initiation (Orpella et al., 2024). As a consequence, rather than facilitating speech fluency, this mechanism could interfere with the ongoing speech motor planning, thereby increasing the likelihood of dysfluencies. As suggested by present findings, this anticipatory process may happen in both simple (i.e., single words) and complex contexts (i.e., sentences).

### 4.2 The role of neuroticism in stuttering

Due of its functions and neural components, the DMN can (with caution) be placed alongside the personality factors of neuroticism (Perkins et al., 2015; Servaas et al., 2014). People with high neuroticism levels usually experience more rumination and worry, thus predisposing to higher activation of DMN nodes (see Fernandes Coutinho et al., 2016; Hamilton et al., 2011; Servaas et al., 2014; Tichenor and Yaruss, 2020; Tseng and Poppenk, 2020).

As reported in the Introduction section, neuroticism is a personality trait characterized by anxiety, anger, depression, self-awareness, impulsivity, withdrawal, volatility, and/or vulnerability. This trait may predict emotional reactivity, that is, the degree and the manner in which a person reacts to specific stimuli, especially to the “negative” ones (Robinson et al., 2013). In fact, subjects with high neuroticism are more likely to change their attitude following errors and are more sensitive to “negative” feedbacks (Robinson et al., 2013). An excessive involvement of the DMN has been reported to have a role in conditions such as depression and social anxiety and, in individuals with major depressive disorder, it may represent the substrate for experiencing higher levels of rumination (Hamilton et al., 2011).

Compatibly, in relation to present findings, it can be suggested that PWS may excessively focus their attentional resources on the inner perspective (vs. the external one, useful for “goal-directed” behavior), also as a consequence of the experienced stuttering anticipation. In this context, when participants have to read a sentence (vs. single-word reading), the increased difficulty of the task also requires greater cognitive, emotional, and motor control over performance, thus further recruiting neural networks such as pre-frontal and anterior cingulate regions (see for example Arnstein et al., 2011; Jackson et al., 2022). Thus, greater cognitive, emotional, and motor demands could increase the likelihood of dysfluencies, especially in an already vulnerable speech motor system, such as in PWS.

Also, neuroticism can influence individual performance: for example, Robinson and Tamir (2005) showed that people with high neuroticism have higher variability of reaction times in different tasks, suggesting that participants with increased neuroticism levels are not slower or faster but they are more inconsistent in their “trial-after-trial” responses (i.e., a finding also associated with possible frontal and/or attentional impairment).

Here, and also based on the above reported evidence, neuroticism seems to interact with stuttering anticipation in modulating speech performance in PWS, especially in the more “complex” and “demanding” contexts (i.e., sentences).

### 4.3 Limitations and future perspectives

A weakness of this study is its sample size (although in line with similar research), predisposing to high “false negatives” (or type II error), as the statistical power of the chosen test may be lower. In this context, increasing sample sizes will be a future goal of our work. Another limitation of the study is the lack of anticipation data for the sentence reading. We opted for using reading times because we evaluated that this index could be a little bit more “stable” with respect to frequency of dysfluencies (however, the presence of a significant positive correlation between dysfluencies and reading times suggested a certain level of “interchangeability” of these measurements in the present work). Finally, the 13 PWS participants overwhelmingly reported having attained at least a bachelor’s degree (84.5% of the sample): this may be unusual compared to other evidence, which may report lower-than-average school and academic success in PWS (O’Brian et al., 2011).

When considering future perspectives, this work suggests the importance of treating stuttering as an extremely complex and wider disturbance, that should be investigated trying to consider also factors such as neuroticism, anxiety sensitivity, and contextual factors such as stuttering anticipation. This highlights the need to enrich research through the use of measures that can evaluate also physiological indices, such as participants’ arousal: this may be a key piece in further understanding mechanisms related to the inefficiency of speech networks when facing particular and/or emotional situations.

If anticipation and neuroticism are in some way involved in stuttering, these should be a therapy target that should be taken into consideration (at least) in preliminary and conclusive moments. In fact, the final goal of stuttering therapy does not only need to bring to an overt fluent speech but also needs to improve the inner feelings, thus trying to eliminate (or accept) internal blocks that should be considered as moments of stuttering themselves.

## 5 Conclusion

In conclusion, we can summarize the findings of this work suggesting that PWS may have a tendency toward higher levels of neuroticism (when compared to normative samples). It can also be suggested that PWS are characterized by social and cognitive concerns, as well as by slower reading times (due to stuttering). Neuroticism (comprising elements such as the depression and the self-consciousness subscales) and stuttering anticipation can effectively predict and explain most of the variance of PWS’ reading times. Among all the factors included as independent variables, stuttering anticipation is the construct that most explains the variance in the models, once again suggesting its importance in stuttering dynamics. The present work also elaborates the anticipation construct: the cognitive-emotional rates of anticipating stuttering on a word, which PWS give in a “low-anxiety” situation (i.e., “pre-task” mode) does not appear to be correlated with the same ratings in “high-anxiety” conditions (i.e., when tested and recorded). However, PWS know when they are about to stutter and they manage with a good deal of accuracy predictions about their “future” fluency in line with increasing anticipation. In this context, regression analyses show that factors such as neuroticism begin to show their effects mainly in “complex” conditions, such as reading sentences. Thus, it seems that the more (linguistically/cognitively/motorically) “demanding” the situation, the more these factors make a negative contribution to speech fluency, also acting as likely maintenance factors.

In conclusion, factors such as stuttering anticipation and neuroticism should be evaluated and considered as possible modulatory factors of dysfluencies in stuttering. As a consequence, they should be actively considered by clinicians when setting up treatments and interventions in DS.

## Data Availability

The raw data supporting the conclusions of this article will be made available by the authors, without undue reservation.
