# Supplementary material for: The role of anticipation and neuroticism in developmental stuttering
Source: Front Psychol. 2025 May 21;16:1576681. doi: 10.3389/fpsyg.2025.1576681 (PMC12133894; doi:10.3389/fpsyg.2025.1576681)
Supplement: Supplementary file 1 [file Table_1.docx]

Supplementary Material

## Supplementary Figures

**
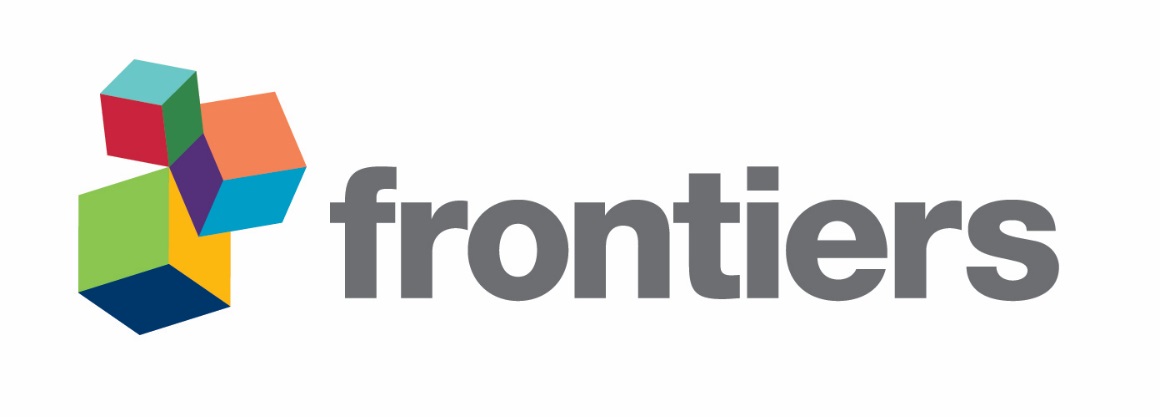
**

**Supplementary Figure 1.** This Figure shows all 55 words used for the experimental task (with the 5 most difficult words of a prototypical participant, in italics).

| Acqua | Donna | Idea | Piatto | Tivù |
| --- | --- | --- | --- | --- |
| Accusa | Ebreo | Ieri | Prego | Trono |
| Ansia | Euro | Labbro | Pronto | Uovo |
| Banca | Età | Lista | Primo | Uscita |
| Brano | Fermo | Molo | Quadro | Zero |
| Bosco | Frutto | Mostro | Quindi | Zona |
| Caffè | Frasca | Nastro | Ritmo | *Grasso* |
| Corpo | Giorno | Niente | Ramo | *Trarre* |
| Cresta | Grazie | Ombra | Scarpa | *Andrea* |
| Danno | Hotel | Onda | Squadra | *Tre* |
| Dentro | Hobby | Pesce | Terno | *Aereo* |

**Supplementary Figure 2.** This figure shows all 55 sentences associated with the 55 words (with the 5 sentences obtained from the most difficult words of a prototypical participant, in italics).

| 1. Buongiorno, gradirei un bicchiere d’acqua per favore. |
| --- |
| 1. Paolo accusa Franca di dargli poche attenzioni. |
| 1. Ho molta ansia per l’esame di lunedì. |
| 1. Devo passare in banca per aprirmi un conto. |
| 1. Ieri ho ascoltato un brano molto bello. |
| 1. Amo veramente andare a passeggiare nel bosco. |
| 1. Buongiorno, gradirei un caffè per favore. |
| 1. Mi piace la sensazione della sabbia sul corpo. |
| 1. Devo andare a tagliarmi i capelli, mi farò la cresta. |
| 1. Dovrò ripagare il danno all’automobile. |
| 1. Dove hai messo le scarpe? “Sono dentro l’armadio”. |
| 1. Il nome “donna” deriva dal latino domina o padrona. |
| 1. Ho letto il libro “L’ebreo errante” e mi è piaciuto molto. |
| 1. Sono a corto di soldi, mi presti 3 euro per fare colazione? |
| 1. Vorrei chiederti l’età, ma non so se ciò risulta scortese. |
| 1. Stai fermo! Hai un insetto addosso, ora te lo tolgo. |
| 1. Il maracuja è considerato il frutto della passione. |
| 1. Il mio prof salta di palo in frasca quando spiega. |
| 1. Buongiorno a tutti, come state oggi? |
| 1. Puoi prestarmi i tuoi occhiali per leggere? Grazie mille. |
| 1. Questa estate abbiamo prenotato un hotel niente male. |
| 1. Il mio hobby preferito è uscire con gli amici. |
| 1. Oggi il datore di lavoro ha tenuto in considerazione la mia idea. |
| 1. Ieri sono andato al mare in Puglia. |
| 1. Vorrei farmi il piercing al labbro ma non ho il coraggio. |
| 1. Prima di andare a fare la spesa dovremmo sempre farci una lista. |
| 1. La nave ha attraccato al molo senza difficoltà. |
| 1. Mia figlia pensa ancora di avere un mostro sotto al letto. |
| 1. Puoi prestarmi del nastro adesivo? |
| 1. Cosa hai fatto ieri? Niente di che, sono uscito un po'. |
| 1. Riesci a fare le ombre cinese con le mani? |
| 1. L’altro giorno ho surfato un’onda gigante. |
| 1. Bisognerebbe mangiare pesce almeno 1 volta a settimana. |
| 1. Ho avuto il piatto più piccolo di tutti. |
| 1. “Grazie tanto di avermi aiutato” “Prego, non c’è di che”. |
| 1. Pronto come posso esserle utile? |
| 1. “Sono arrivato primo alla competizione”. |
| 1. Adoro il quadro di Van Gogh la “Notte stellata”. |
| 1. Ho fatto un ottimo lavoro, quindi sono stato rinnovato. |
| 1. Il ritmo della musica mi mette sempre tanta allegria. |
| 1. Vorrei andare a leggere sotto quel ramo. |
| 1. Ho comprato una scarpa troppo piccola. |
| 1. Nelle attività mi piace giocare di squadra. |
| 1. Con te è sempre un terno a lotto! |
| 1. Questo fine settimana voglio solo guardare la tivù. |
| 1. Il trono di spade è la mia serie preferita. |
| 1. Il mio piatto preferito è l’uovo in camicia. |
| 1. Prego signori l’uscita è sulla vostra sinistra. |
| 1. Oggi ho zero voglia di andare al lavoro. |
| 1. Oggi passo nella tua zona, se vuoi ci incontriamo. |
| 1. *Il grasso è la parte più buona del prosciutto.* |
| 1. *Bisogna sempre trarre il meglio dalle cose.* |
| 1. *Mio figlio si chiama Andrea e ha 22 anni.* |
| 1. *Tre a zero è il risultato di Juventus-Inter* |
| 1. *Ho molta paura dell’aereo! Non riesco proprio a prenderlo* |
